# Histological and Histomorphometric Evaluation of Applying a Bioactive Advanced Platelet-Rich Fibrin to a Perforated Schneiderian Membrane in a Maxillary Sinus Elevation Model

**DOI:** 10.3389/fbioe.2020.600032

**Published:** 2020-11-26

**Authors:** Liangjing Xin, Shuai Yuan, Zhixiang Mu, Dize Li, Jinlin Song, Tao Chen

**Affiliations:** Chongqing Key Laboratory of Oral Diseases and Biomedical Sciences, Chongqing Municipal Key Laboratory of Oral Biomedical Engineering of Higher Education, Stomatological Hospital of Chongqing Medical University, Chongqing, China

**Keywords:** Schneiderian membrane, advanced platelet-rich fibrin, collagen membrane, perforation, animal models

## Abstract

**Background:**

Schneiderian membrane (SM) perforation is a major complication of maxillary sinus elevation with simultaneous bone grafting, yet under this scenario there is no standard biomaterial that maximizes favorable tissue healing and osteogenic effects.

**Purpose:**

To compare the effect of advanced platelet-rich fibrin (A-PRF) and collagen membrane (CM) on a perforated SM with simultaneous bone grafting in a maxillary sinus elevation model.

**Materials and Methods:**

After perforation of the SM was established, 24 animals were randomly divided into two groups: (i) group CM: CM and deproteinized bovine bone mineral (DBBM) (*n* = 12), (ii) group A-PRF: A-PRF and DBBM (*n* = 12). Radiographic and histological evaluations were performed at 1 and 4 weeks post-operation.

**Results:**

At 1 week, an intact SM was found in group A-PRF. At each time point, the number of inflammatory cells at the perforated site was higher in group CM, and the area of new osteoid formation was significantly greater in group A-PRF (*p* < 0.0001). At 4 weeks, the osteogenic pattern was shown as from the periphery to the center of the sinus cavity in group A-PRF.

**Conclusion:**

The higher elasticity, matching degradability, and plentiful growth factors of A-PRF resulted in a fully repaired SM, which later ensured the two osteogenic sources from the SM to generate significant new bone formation. Thus, A-PRF can be considered to be a useful bioactive tissue-healing biomaterial for SM perforation with simultaneous bone grafting.

## Introduction

Long-term loss of the maxillary posterior teeth often leads to a series of complications, such as maxillary sinus pneumatization and ridge atrophy. These conditions may increase the implant failure rate (Asai et al., 2002; Stricker et al., 2003; Wallace and Froum, 2003; Sorní et al., 2005; Tajima et al., 2013). To manage unfavorable results, clinicians often adopt maxillary sinus floor elevation to increase the bone volume of the atrophic maxilla and ensure successful implant placement (Schwartz-Arad et al., 2004; Hallman et al., 2005).

However, despite accurate preoperative radiographic investigations and surgical maneuvers, Schneiderian membrane (SM) perforation can occur during the elevation process, and it has a reported incidence of 56% (Aricioglu et al., 2017). Irregular morphology of the maxillary sinus and the fragile characteristics of the SM contribute to this unfavorable outcome (Schwartz-Arad et al., 2004; Misch and Wang, 2008; Schwarz et al., 2015; Wen et al., 2015). The perforation of the SM may lead to severe complications including the suspension of a surgical process, acute maxillary sinusitis, and an unpredictable survival rate of the dental implants (Cho et al., 2001; Timmenga et al., 2003; Proussaefs et al., 2004; Anavi et al., 2008; Becker et al., 2008).

The treatment of the perforation during sinus elevation depends on the perforation size. When the perforation is less than 5 mm (Hernández-Alfaro et al., 2008), the most common repair procedure is to use an absorbable collagen membrane (CM), which minimizes the risk of infection and usually achieves satisfactory clinical results (Aimetti et al., 2001; Proussaefs et al., 2004; Ardekian et al., 2006; Pikos, 2008). Lim et al. (2018) confirmed that the absorbable CM greatly reduced infection in the sinus cavity and could be used as repair material for SM perforation.

Nevertheless, the dense structure of the CM might block the osteogenesis of the SM. The SM is a possible source of osteogenesis in the maxillary sinus (Srouji et al., 2009; Jung et al., 2015; Mu et al., 2020), and the implantation of an absorbable CM might slow new bone formation in the sinus cavity (Gruber et al., 2004; Palma et al., 2006). Although CMs have been widely used to repair SM perforation, it is not clear if the degradation and mechanical properties of the CM are compatible with the repair process. In addition, its high cost and potential foreign body reaction caused by its porcine sources are issues (Schorn et al., 2019).

Platelet-rich fibrin (PRF), as a self-clotted preparation of platelet-concentrated and autologous blood–derived biomaterial, has been advocated in several studies and produced favorable outcomes for SM perforation during maxillary sinus floor elevation (Panda et al., 2014; Zhao et al., 2015). Advanced PRF (A-PRF) is one of several PRF derivatives, produced by a relatively lower speed centrifugation process (Aizawa et al., 2020). Because of this specific preparation process, the three-dimensional fibrin matrix is more porous than that of the original PRF, and more growth factors, leukocytes, and platelets are “trapped” in its fibrin matrix structure (Lundquist et al., 2008; Ghanaati et al., 2014; Nishimoto et al., 2015; Takeda et al., 2015; Masuki et al., 2016). The trapping ensures that significant amounts of growth factors are present and slowly release (Choukroun et al., 2006; Masuki et al., 2016; Isobe et al., 2017). The fibrin matrix with porous structure could mimic the extracellular matrix, creating an optimal environment for cell adhesion and migration (Choukroun et al., 2006; Roy et al., 2011; Pradeep et al., 2012; Ghanaati et al., 2014; Aricioglu et al., 2017). These studies suggest that A-PRF would function not only as a physical membrane for the perforated site but also as a bioactive tissue-healing “factory” to deliver growth factors for soft and hard tissue repair.

Although an animal study (Aricioglu et al., 2017) has demonstrated that PRF could be used as a substitute for CM in repairing SM perforations, no previous studies have focused on the repair capabilities of CM and A-PRF regarding simultaneous bone grafting. We therefore evaluated the effectiveness of A-PRF and CM for SM repair with bone grafting simultaneously in a rabbit model.

## Materials and Methods

### Preparation of A-PRF

A-PRF was prepared as previously described (Aizawa et al., 2020). Briefly, 9 mL of autologous blood was taken from the central ear artery of a rabbit and collected into tubes (Plain BD Vacutainer Tube; Becton, Dickinson and Company, Franklin Lakes, NJ, United States) free from anticoagulant. The samples were produced using a programmed Duo Quattro centrifugation system (Process for PRF, Nice, France) with 200 × *g* for 14 min. Then, three layers emerged in the anticoagulant-free tube. Acellular plasma was separated, and the red blood cells attached to the A-PRF were removed with a knife (Supplementary Figure S1A). After eliminating the red blood cells, A-PRF was compressed to a thin film (Supplementary Figure S1B) using a compression device (the PRF Box, Process, Nice, France), as shown in Supplementary Figure S1C.

### Characterization of CM and A-PRF

#### Scanning Electron Microscopy

CM and A-PRF were both fixed with 2.5% neutralized glutaraldehyde, dehydrated with a series of ethanol solutions and t-butanol, freeze-dried, and then examined under a scanning electron microscope (SEM) (FEI, Quanta 450, United States) with an accelerating voltage of 15 kV.

#### Mechanical Testing

The compressed A-PRF, moist CM, and the natural SM were selected for mechanical testing. All membranes were cut into 20 × 5-mm (length × width) rectangular strips for mechanical testing of elasticity. The mechanical properties of different membranes were measured at a stretching speed of 1 mm/min with a desktop universal testing machine (E43, MTS Instrument, United States), where the maximum load cell capacity was 100 N under standard ambient conditions at 25°C ± 3°C and 50 ± 25% relative humidity (RH). The elastic modulus was defined as the average slope of the initial part (0–10% strain) of the stress–strain curve.

#### Animal Model

Animal experiment protocols were approved by the Animal Ethics Committee of Chongqing Medical University (CQHS-IRB-2018-07) and were conducted according to National Institutes of Health guidelines. We used 24 male New Zealand rabbits with weights ranging from 3 to 3.5 kg. After the SM perforation was established, rabbits were randomly divided into two groups: (i) group CM: CM and deproteinized bovine bone mineral (DBBM) (*n* = 12), (ii) group A-PRF: A-PRF and DBBM (*n* = 12). The effect of each group (*n* = 6, respectively) was assessed at two healing time points that were 1 and 4 weeks post-operation.

#### Animal Surgery

**FIGURE 1:**
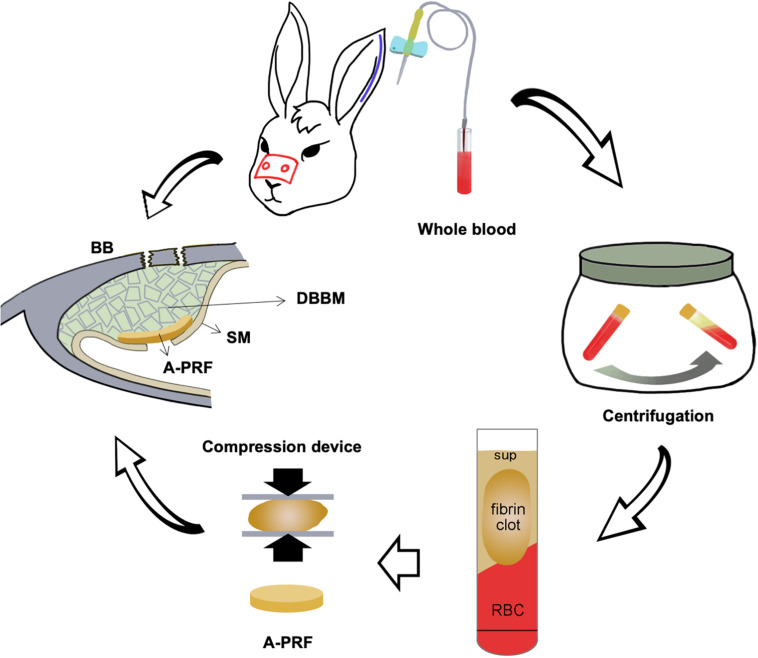
Schematic illustration of placing an A-PRF on a perforated SM in a rabbit maxillary sinus elevation model. DBBM, deproteinized bovine bone mineral; BB, basal bone; RBC, red blood cell; Sup, supernate; SM, Schneiderian membrane; A-PRF, advanced platelet-rich fibrin.

**FIGURE 2:**
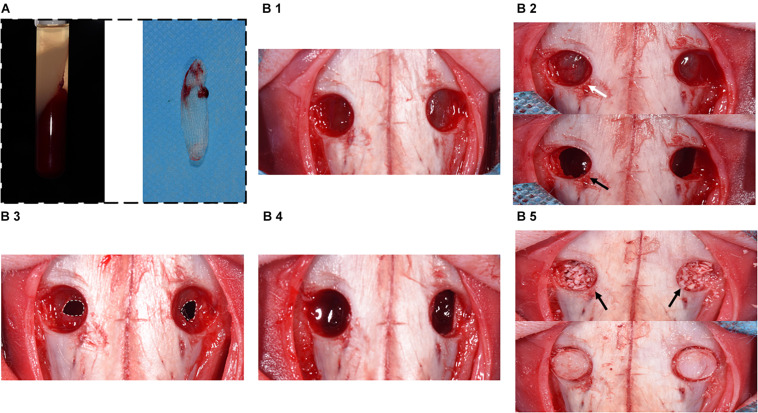
Surgical procedure diagram in a perforated SM model. **(A)** Preparation of A-PRF. **(B1)** Symmetrical bone defects were obtained, and bone plates were acquired correspondingly. **(B2)** SM was detached and elevated from bony walls (marked with white arrow). Rhythmic movement of SM during respiration (black arrow). **(B3)** SM was perforated with a 1-cm incision (marked with the white dotted box). **(B4)** CM or A-PRF was placed onto the perforated SM. **(B5)** The sinuses were filled with DBBM (marked with black arrows). Finally, the bone defects were covered with bone plates.

The placement of the materials on the perforated SM is illustrated in Figure 1. All operations were performed under sterile conditions by one surgeon (Liangjing Xin). The 24 rabbits were subjected to the maxillary sinus floor elevation process as previously described (Mu et al., 2020). In detail, the rabbits were anesthetized using 30 mg/kg xylazine hydrochloride (Rompun; Bayer, Seoul, Korea). Surgical sites were shaved and disinfected with an iodine solution. Local anesthesia was used to minimize pain at the surgical site using 2% lidocaine HCl (20 mg/kg; Huons, Sungnam, Korea). An incision was made from the nose to eye level to expose the nasal bone. Symmetrical bone defects were created using a circular drill (drill diameter = 5 mm), and the bone plates were removed. Entering through these openings, the SM was detached and elevated from the bony walls. Afterward, a perforation was made using a blade in a sagittal direction (perforation diameters = 3 mm/half of the extension of osteotomy; Lim et al., 2018). In group CM, an absorbable CM (Bio-Gide; Geistlich Pharma, Wolhusen, Switzerland) was cut into a 10 × 10-mm section and placed onto the perforated SM, extending onto the lateral and medial sinus bone walls. Autologous blood was taken, and A-PRF was obtained according to the protocol above. A-PRF was compressed and cut into 10 × 10-mm pieces and then placed onto the SM perforation correspondingly. All sinus cavities were grafted using a standardized amount (0.2 cc) of DBBM (Bio-Oss; Geistlich Pharma) (Figure 2). Finally, the bone defect was covered with a bone plate, and the wound was closed using absorbable monofilament (Vicryl 5-0; Ethicon, MA, United States).

Animals in the 4-week groups were subcutaneously injected with tetracycline (TE, 25 mg/kg; Sigma, St. Louis, MO, United States), calcein (CA, 25 mg/kg; Sigma), and alizarin complexone (AL, 30 mg/kg; Sigma) at the first, second, and third week post-operation, respectively, to observe the osteogenic patterns.

The rabbits were monitored, and antibiotics and analgesics were administered on the first 3 d post-operation.

#### Sacrifice and Sample Collection

At the 1- and 4-week healing time points, rabbits were euthanized by injection of sodium pentobarbital (Sigma-Aldrich, St. Louis, MO, United States) through the central ear artery. The maxillary sinus samples were collected and processed for micro–computed tomographic (micro-CT) analysis and histological evaluation.

### Micro–Computed Tomographic Analysis

The 4-week post-operation maxillary sinus samples were analyzed using a micro-CT (vivaCT80; SCANCO Medical AG, Switzerland). The scanning condition was acquired at a resolution 14.91 μm (130 kV and 60 μA). Three-dimensional reconstruction of the interest areas was performed withμCT80 (SCANCO Medical AG).

### Histological Analysis

Half of the specimens in the 4-week groups were obtained and dehydrated in a graded series of ethanol and embedded in methyl methacrylate (M55909; Sigma). Subsequently, the specimens were prepared using the Hard Tissue Sawing System (E200CP; EXAKT Verteriebs, Germany). Tissue slices were observed with a fluorescent microscope (Olympus, Tokyo, Japan) for fluorescent labeling. Finally, the samples were stained with Van Gieson (VG). In addition, the 12 specimens at 1 and 4 weeks post-operation were decalcified and embedded in paraffin. They were then stained with Picro-Sirius red stain for observation of re-epithelialization during SM repair. Hematoxylin-eosin (H&E) staining was used to evaluate early and later inflammatory responses. Aniline blue and osteocalcin immunohistochemical staining (IHC) were also used to assess formation of new bone. Osteoclast activity was observed using tartrate-resistant acid phosphatase (TRAP) staining.

### Histomorphometric Analysis

The tissue sections showed that the fracture of the basal bone corresponded to the perforated area of the SM (Supplementary Figures S2A,B), and the regions of interest (ROIs) were under the perforated SM area (ROIa) and underneath the CM and A-PRF (ROIb). Histomorphometric calculation of tissue sections, which were stained with H&E, aniline blue, IHC, and TRAP, was conducted using a Olympus Research System Microscope BX51 (Olympus). The healing patterns of the perforated SM were analyzed. For the stained images, histomorphometric analysis included the following parameters:

• Relative proportion of different cells (%): the percentage of pixels (inflammatory cells, fibroblasts, eosinophils) in the ROIa according to H&E staining.
• The area of new osteoid formation (%): blue-stained mineralized tissue area including osteocytes with the use of aniline blue staining, which is described as a percentage of the whole ROI for semiquantitative analysis (ROIa at 4-week groups, ROIb at 1-week groups).
• Relative expression of osteocalcin (%): percentage of pixels associated with deeply stained osteocalcin-positive cells in the ROIa by IHC staining.
• The relative expression of osteoclasts (%): osteoclasts are recognized as the TRAP-positive cells with the use of TRAP staining sections, which are expressed as a percentage of the ROI for semiquantitative analysis (ROIa at 4-week groups, ROIb at 1-week groups).

### Statistical Analysis

All data had a normal distribution and were analyzed using SPSS Statistics version 20.0 (IBM Corp., Armonk, NY). Statistical analysis was performed by Student *t*-tests using GraphPad Software v6 (GraphPad Software, La Jolla, CA, United States), and statistical significance was considered at *p* < 0.05. All data are expressed as the mean ± standard deviation.

## Results

### Clinical Findings

All experimental animals undergoing surgical procedures maintained a healthy status throughout the entire experimental period. No complications were observed during the postoperative period.

### Characterization of CM and A-PRF

**FIGURE 3:**
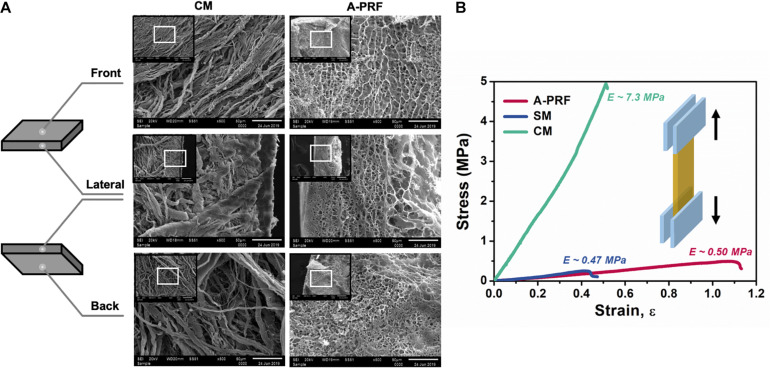
The characterization of CM and A-PRF. **(A)** Representative microstructural images of the freeze-dried CM and A-PRF at different layers (scale bar = 50 μm). **(B)** The representative stress–strain curves of tensile test on CM, A-PRF, and the natural SM. CM, collagen membrane; A-PRF, advanced platelet-rich fibrin; SM, Schneiderian membrane.

Microstructures of CM and A-PRF were examined by SEM. As shown in Figure 3A, the fibrin matrix within A-PRF was thicker and denser than that within the CM. In the cross section, the CM microstructure showed two different layers. Most of the collagen fibers in the front layer were close to each other, indicating a low porosity with a smooth surface. The back layer showed an uneven distribution of collagen fibers, which had a more porous appearance with a rough surface. Collagen fibers were also larger in diameter and arranged in bundles. Nevertheless, both the front and back layers of A-PRF showed reticular and porous microstructures, which were significantly softer and looser than those of CM. The fibronectin was slender and staggered in A-PRF.

Tensile properties among CM, A-PRF, and the natural SM are shown in the stress–strain plot (Figure 3B). The yield strain was about 51.2 ± 0.1% in CM, 109.7 ± 0.3% in A-PRF, and 43.3 ± 0.2% in the natural SM. The yield strain in A-PRF was significantly higher than that in CM (*p* < 0.0001), indicating that A-PRF had superior elasticity.

### Histological Analysis

**FIGURE 4:**
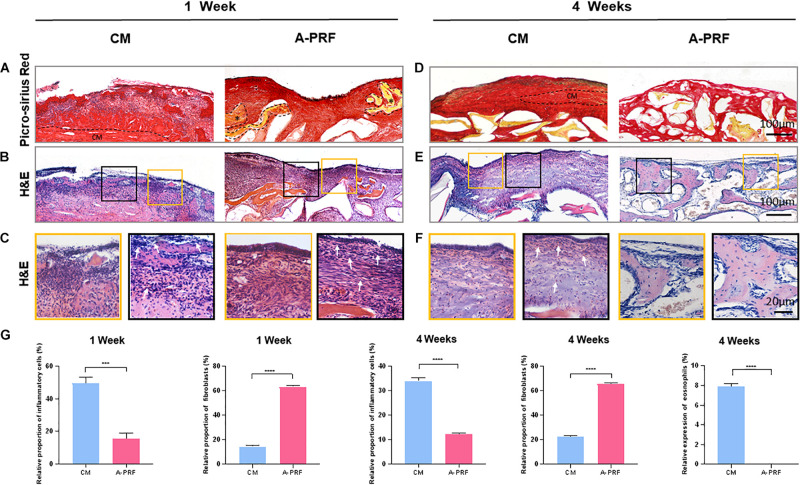
Histological and histomorphometric analysis of soft tissue healing under the perforated SM at 1 and 4 weeks. **(A)** Picro-Sirius red staining on group CM and A-PRF at 1 week post-operation (A-PRF marked with *). **(B)** H&E staining at 1 week post-operation. **(C)** Two ROIs (yellow box and black box) selected to further observe the microscopic components of **(B)**. **(D)** Picro-Sirius red staining at 4 weeks (the dotted box represented the residual CM). **(E)** H&E staining evaluating later inflammatory responses in each group. **(F)** Two ROIs (yellow box and red box) of **(E)**. **(G)** Semiquantitative analysis of the inflammatory reaction at 1 and 4 weeks by measuring the proportion of inflammatory cells, fibroblasts and eosinophils (*n* = 3, ***p < 0.001, ****p < 0.0001). CM, collagen membrane; A-PRF, advanced platelet-rich fibrin; ROIs, regions of interest.

A dome-shaped space was observed in the elevated maxillary sinus in two groups (Supplementary Figures S2A,B). The DBBM was well-distributed within the sinus cavity, and the fracture of the basal bone corresponded exactly to the perforated area. A-PRF was not observed at the repaired site, while CM was found intact under the SM (Figure 4A).

H&E staining (Figure 4B) revealed an intact SM in group A-PRF, showing that, at the perforated site, a pseudostratified columnar ciliated epithelium facing the sinus cavity comprised a plentiful vascularized lamina propria and a deeper layer of periosteum-like components (Figure 4C, yellow box); however, this structure was not observed in group CM. In addition, the aggressive infiltration of inflammatory cells (marked with white arrows) was dispersed (Figure 4C, black box), suggesting that a severe inflammatory response occurred under the perforated SM in group CM. An increasing number of newborn fibroblasts (spindle-shaped or flat star-shaped with protrusions, marked with white arrows) occurred in group A-PRF at 1 week post-operation (Figure 4C, black box). This indicated that the early inflammation stage was replaced by the tissue repair process.

Micro-CT (Supplementary Figures S2C1–2) reconstruction was performed to simulate the sinus cavity at 4 weeks post-operation. No leakage of DBBM was observed, and the perforated site was completely repaired.

As shown by Picro-Sirius red staining at 4 weeks post-operation (Figure 4D), the CM did not completely degrade, and the newly reconstructed mucosa significantly thickened in group CM because of the inflammatory response (the dotted box represents the residual CM in Figure 4D). However, in group A-PRF, the perforated SM repaired with A-PRF was completely degraded. To study the internal mechanism of this phenomenon, H&E staining (Figure 4E) was performed on 4-week sections. At high magnification (Figure 4F), pseudostratified columnar ciliated epithelium was not observed (yellow box), and a large amount of inflammatory cell infiltration (black box) was observed underneath the SM in group CM. Eosinophils (typical lobulated nuclei, containing eosinophilic granules in the cytoplasm, marked with white arrows) and inflammatory cells formed an infiltration zone surrounding residual non-degraded CM (black box). In contrast, the newly repaired mucosa resembled the natural SM (yellow box) and had a large number of fibroblasts (black box) in group A-PRF.

**FIGURE 5:**
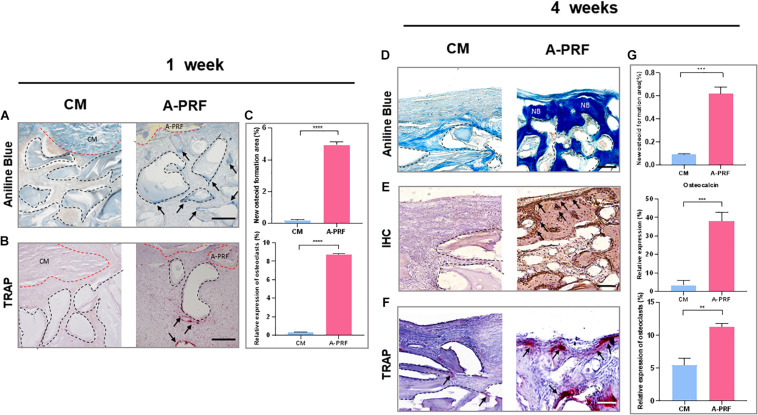
Histological and histomorphometric analysis of new bone formation under SM area at 1 and 4 weeks. **(A)** Aniline blue staining for assessing new bone formation in group CM and A-PRF at 1 week (blue-stained mineralized tissues were marked with black arrows; black dotted boxes represented DBBM, scale bar: 100 μm). **(B)** TRAP staining for the osteoclast activity at 1 week (TRAP-positive cells were marked with black arrows, scale bar: 100 μm). **(C)** Semiquantitative analysis regarding new osteoid formation and the relative expression of osteoclast area at 1 week (*n* = 3, ****p < 0.0001). Aniline blue staining **(D)**, IHC **(E)**, and TRAP staining **(F)** were performed to further reveal bone remodeling in group CM and A-PRF at 4 weeks (scale bar: 50 μm). **(G)** Semiquantitative analysis regarding new osteoid formation, relative expression of osteocalcin, osteoclast area at 4 weeks post-operation (*n* = 3, **p < 0.01, ***p < 0.001). CM, collagen membrane; A-PRF, advanced platelet-rich fibrin; DBBM, deproteinized bovine bone mineral; NB, new bone.

Based on aniline blue staining (Figure 5A), there was little blue-stained mineralized tissue underneath the CM, which showed the least amount of new bone formation at 1 week in group CM. In contrast, the representative histological sections (black arrows) underneath A-PRF revealed a small amount of new bone formation in group A-PRF at an early stage. In TRAP staining (Figure 5B), few osteoclasts were seen around the DBBM at 1 week in group CM. However, a few osteoclasts that infiltrated around the DBBM occurred in group A-PRF. Aniline blue staining at 4 weeks post-operation (Figure 5D) showed little new bone formation in group CM, while evident blue-stained mineralized tissue was observed under the SM in group A-PRF.

In IHC, group A-PRF exhibited strongly osteocalcin-positive cells under the SM, whereas almost no positive expression was shown in group CM (Figure 5E). In TRAP staining at 4 weeks (Figure 5F), little TRAP-positive cell expression and low osteoclast activity were observed under the SM in group CM. In contrast, many more osteoclasts were detected underneath the SM, suggesting active bone remodeling in group A-PRF.

**FIGURE 6:**
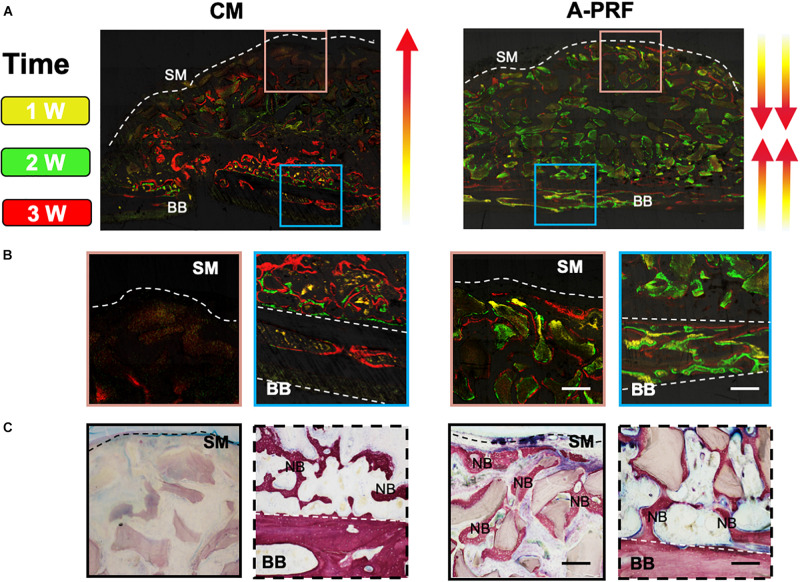
Osteogenic patterns in group CM and group A-PRF at 4 weeks. **(A)** Fluorescent labeling (tetracycline shown as yellow, calcein as green and alizarin red red) of the two groups. **(B)** Magnified images of two ROIs indicating the direction of new bone formation: (1) area underneath the SM (pink box); (2) area near the basal bone (blue box). **(C)** VG staining at 4 weeks. SM, Schneiderian membrane; BB, basal bone; NB, new bone (scale bar: 100 μm).

While distinguishing the osteogenic patterns in each group, a fluorochrome label of new bone mineralization was detected. Weak fluorescence signals were observed in group CM, whereas group A-PRF displayed stronger fluorescence intensity (Figure 6A). The fluorescence signals were randomly scattered in the basal bone in group CM (Figure 6B, blue box). However, the three-color fluorescence signals were distributed both in the basal bone (Figure 6B, blue box) and underneath the SM (Figure 6B, pink box) in group A-PRF. VG staining (Figure 6C) revealed a large amount of new bone formation near the basal bone in the two groups, whereas the new bone growth was generated from the periphery to the center of the sinus cavity in group A-PRF.

### Histomorphometric Analysis

The relative proportion of inflammatory cells was significantly different between group CM and A-PRF (49.37 ± 3.83% vs. 15.43 ± 3.44% at 1 week, *p* < 0.001; 33.87 ± 1.29% vs. 12.23 ± 0.25% at 4 weeks, *p* < 0.0001). The relative proportion of fibroblasts in group A-PRF was measured compared to group CM, which was significant (63.37 ± 1.19% vs. 14.43 ± 1.19% at 1 week, 65.7 ± 0.9% vs. 22.33 ± 1.12% at 4 weeks, *p* < 0.0001). Eosinophils in group A-PRF were significantly fewer than in group CM (0% vs. 7.9 ± 0.3% at 4 weeks, *p* < 0.0001) (Figure 4G).

At both healing time points, the percentage of new osteoid formation was significantly greater in group A-PRF compared to group CM (4.93 ± 0.21% vs. 0.18 ± 0.06% at 1 week, *p* < 0.0001; 0.62 ± 0.06% vs. 0.09 ± 0.01% at 4 weeks, *p* < 0.001) (Figure 5C). The relative expression of osteocalcin at 4 weeks was significantly different in group A-PRF (37.93 ± 4.91%) and group CM (3.33 ± 2.63%) (*p* < 0.001). In addition, there was a significant difference between group A-PRF and group CM in the relative expression of osteoclasts (8.72 ± 0.06% vs. 0.32 ± 0.01% at 1 week, *p* < 0.0001; 11.27 ± 0.5% vs. 5.43 ± 1.12% at 4 weeks, *p* < 0.01) (Figure 5G).

## Discussion

Perforation of the SM occurs in the maxillary sinus elevation at a frequency of 10–56% (Pikos, 2008; Nolan et al., 2014; Shiffler et al., 2015; Aricioglu et al., 2017). Although an absorbable CM has been proposed, there is no standard treatment for the repair of SM perforation when the size is less than 5 mm. Most of the following qualities should be present in a proper tissue-healing membrane regarding perforated SM (Al-Maawi et al., 2019): (a) appropriate mechanical properties allowing combination with natural tissue and providing an intact microenvironment for tissue remodeling; (b) suitable degradative profile matching the neotissue formation; (c) non-immunogenicity allowing integration of the membrane with the host tissue without triggering an overinflammatory effect; (d) being rich in cells and growth factors to provide a bioactive basis through biomaterial-induced tissue reactions.

A CM is a double-layered absorbable barrier membrane that has been widely used in guided bone/tissue regeneration (GBR/GTR) (Rothamel et al., 2005; Dimitriou et al., 2012; Al-Maawi et al., 2019; Schorn et al., 2019). The back layer is exposed to the bony defect, allowing osteogenic cells to immigrate to the repair site, while the front layer is exposed to the soft tissue and used to prevent soft tissue ingrowth (Lang et al., 1994; Schorn et al., 2019). According to SEM images (Figure 3A), the large bundles of collagen fibers within the bilayered CM were arranged in a parallel horizontal direction. In contrast, A-PRF was a reticular structure composed of fibronectin. The mechanical properties of barrier membranes are largely related to their microstructure, and proper mechanical properties can facilitate favorable tissue repair (Ghanaati et al., 2014; Masuki et al., 2016; Fujioka-Kobayashi et al., 2017). As shown in the stress–strain curve (Figure 3), yield strain was significantly higher in A-PRF than that in CM (109.7 ± 0.3% vs. 51.2 ± 0.1%, *p* < 0.0001), suggesting that the superior elasticity shown in A-PRF was due to its reticular and porous microstructure. Therefore, the superior elasticity of A-PRF avoided secondary perforation caused by breathing movement and overfilling of DBBM in the repair site, which provided a good foundation for perforated SM repair.

As a physical barrier in GBR/GTR, CM maintains its integrity to promote bone tissue ingrowth (Chu et al., 2017). Premature resorption of the CM will cause tissue regeneration failure (e.g., soft tissue ingrowth) and produce a longer treatment period. In contrast, for tissue-healing biomaterials implanted *in vivo*, timely degradation and appropriate immunogenic characteristics are important prerequisites to facilitate the repair process. As shown in Figure 4A, there was a crevice in the SM in group CM, while continuous and complete repair of the SM in group A-PRF and A-PRF was not observed at the repair site. These outcomes suggest that the CM was less prone to degradation due to the dense collagen fiber network, whereas the degradation of A-PRF and repair of the perforated SM occurred simultaneously for the porous microstructure of A-PRF. Although CM has been the preferred clinical choice for GBR/GTR, it has limitations in the process of perforated SM repair. In addition, H&E staining (Figure 4B) revealed an intact repaired SM in group A-PRF, showing a pseudostratified columnar ciliated epithelium at the perforated site (Figure 4C, yellow box). This structure was similar to a natural SM previously reported (Srouji et al., 2009) and demonstrates the satisfactory tissue repairing ability of A-PRF as applied to a perforated SM. Based on histological images (Figure 4C, black box), the number of inflammatory cells in group A-PRF was significantly lower than that in group CM (15.43 ± 3.44% vs. 49.37 ± 3.83%, *p* < 0.001), whereas the fibroblasts were greatly increased compared to group CM at an early stage (63.37 ± 1.19% vs. 14.43 ± 1.19%, *p* < 0.0001) (Figure 4G). After *in vivo* implantation of biomaterials, acute inflammation occurs following the initial host–material interaction. This leads to a neutrophil influx at the interface of the perforated SM biomaterials. The neutrophils secrete enzymes to degrade the biomaterials and release chemokines and cytokines to recruit and activate monocytes. The monocytes differentiate into macrophages to enhance their phagocytosis. As a foreign biomaterial, porcine-derived CMs will inevitably trigger host–membrane immune response after implantation, which involves the activation of phagocytic cells. The cell-mediated degradation may be involved in the CM degradation process (Fang et al., 2020), and this overinflammatory state produces an adverse microenvironment for tissue repair. Nevertheless, a cocktail of growth factors with A-PRF, such as transforming growth factor β (TGF-β), platelet-derived growth factor (PDGF), and vascular endothelial growth factor (VEGF), can actively trigger and orchestrate the tissue repair processes (Marx, 2004; Aminabadi, 2008; Peerbooms et al., 2010; Soloviev et al., 2014; Herath et al., 2018). Specifically, TGF-β could regulate macrophage polarization from M1 to M2 phenotypes, which eventually reaches more cells of the tissue repair brigade (Dohan et al., 2006; Nasirzade et al., 2020). Additionally, A-PRF could modulate the inflammatory responses by the nuclear factor κB signal pathway (Nasirzade et al., 2020). Therefore, because of the rich growth factors and non-immunogenic characteristics of A-PRF, a continuous and intact pseudostratified columnar ciliated epithelial structure formed at the perforated site without triggering overinflammation. This provided a proper base for tissue repair at an early stage. At 4 weeks post-operation, an intact pseudostratified columnar ciliated epithelium was not observed in group CM (Figure 4F, yellow box). The residual CM was still observed (Figure 4D), and an increasing number of inflammatory cells (33.87 ± 1.29%) and eosinophils (7.9 ± 0.3%) appeared under the perforated SM area. This persistent inflammatory state resulted in a persistent allergic reaction (Figure 4F, black box) and unfavorable mucosa thickening (Figure 4F, yellow box). The perforated SM had been fully integrated, and the A-PRF completely degraded in group A-PRF (Figure 4). Overall, the intact microenvironment created by A-PRF with substantial cells and growth factors served as a bioactive barrier through favorable biomaterial-induced tissue reactions for the timely degradation and non-immunogenic characteristics of A-PRF.

SM repair restores the integrity of the SM and also establishes a microenvironment suitable for new bone formation and remodeling after the maxillary sinus floor elevation process. Kuchler et al. (2020) concluded that DBBM cannot perform the function of creeping substitution in an inflammatory microenvironment. However, because of the formation of the overinflammatory microenvironment in group CM, histological examinations at 1 week (Figure 5A) showed significantly lower new bone formation (0.18 ± 0.06%), and TRAP staining (Figure 5B) revealed lower osteoclast activity (0.32 ± 0.01%) in group CM. Such an overinflammatory microenvironment might hinder the physiological functions of osteoblasts–osteoclasts and delay bone remodeling. Thus, the creeping substitution process could not be operated as scheduled in group CM. In contrast, early osteogenesis began to occur under the SM in group A-PRF (Figures 5A,B). As previously noted, bioactive factors in A-PRF suppress inflammation, and the low-inflammatory microenvironment favored continuous self-renewal of the sinus cavity in group A-PRF (Nasirzade et al., 2020; Zhang et al., 2020) and reached a dynamic balance between bone formation and resorption. In addition, the significant amount of growth factors and cytokines within A-PRF also played an essential role in regulating the bone remodeling process (Marx, 2004; Aminabadi, 2008; Peerbooms et al., 2010; Soloviev et al., 2014; Herath et al., 2018). Growth factors (such as PDGF-BB and VEGF) in A-PRF could stimulate neovascularization, which is essential for osteoblasts to promote osteogenic differentiation (Fernández-Barbero et al., 2006). In group A-PRF, we also observed a large amount of new bone formation (0.62 ± 0.06%) and an increasing number of osteoclasts (11.27 ± 0.5%) under the SM, which demonstrated an active creeping substitution and bone reconstruction process (Figures 5D–F).

Based on previous studies, there are two sources of osteogenesis in the elevated sinus floor area. One is osteogenesis from the basal bone, and the other is from the SM (Srouji et al., 2009; Mu et al., 2020). Figures 6B,C show that the osteogenic pattern of the CM originated solely from the basal bone. However, the dense CM structure caused untimely degradation, which hindered repair of the perforated SM. Even though the CM prevented the ingrowth of soft tissue in GBR/GTR, the residual CM simultaneously blocked one of the osteogenic sources in the sinus cavity. As a result of the closure of the SM (Figures 6B,C), the presence of A-PRF established an intact microenvironment with low inflammation that was conducive to bone formation and remodeling. Because of its rich growth factors and matching degradation, and the two osteogenic sources as mentioned above, newly formed bone was induced to grow along both the basal bone to SM and the SM to basal bone directions.

This study revealed that (i) significant SM repair occurred when utilizing A-PRF, and the degradation of A-PRF was matched with the SM repair process at an early stage; (ii) bone remodeling in the sinus cavity was active, and a greater amount of new bone formation occurred under the perforated SM area in the A-PRF group at a later time point. This is the first preclinical study evaluating A-PRF as an alternative to CM for repair of SM perforation with the filling of DBBM simultaneously.

Several clinical studies investigated a potential benefit due to the placement of a blood product membrane to the perforated SM (Oncu and Kaymaz, 2017; Malzoni et al., 2020). Those clinical studies aimed to evaluate the effect of applying a blood product membrane to SM perforation on osseointegration and the survival rate of dental implants. Although there were several published studies using the blood product membrane, studies evaluating the efficacy of A-PRF in the repair of SM perforations regarding simultaneous bone grafting are lacking. In the present study, we have investigated the efficacy of the healing process of perforated SM and osteogenic pattern through histological and histomorphometric evaluation.

The rabbit experimental model was first introduced by Watanabe et al. (1999) to mimic a perforated SM in a human maxillary sinus elevation procedure. The rabbit maxillary sinus cavity is appropriate for maxillary sinus elevation, as the sinus cavity communicates with the nasal cavity through a well-defined ostium (Kim et al., 2012). Although the rabbit sinus cavity shows similarities to the human maxillary sinus, it differs in the number of platelets. Coagulation factors in rabbit blood are more abundant than in human blood, making the healing pattern presented in the rabbit faster than that which occurs in humans (Butterfield et al., 2005). However, because of ethical issues, this procedure has not been adopted clinically. An additional clinical trial, with a larger simple size and a longer time point, should be conducted to verify the effectiveness of A-PRF.

## Data Availability Statement

The raw data supporting the conclusions of this article will be made available by the authors, without undue reservation.

## Ethics Statement

The animal study was reviewed and approved by the Animal Ethics Committee of Chongqing Medical University (CQHS-IRB-2018-07).

## Author Contributions

LX: design, methodology, data analysis, drafting article, validation, animal experiments, and data collection. SY: critical revision of article, statistics, and approval of article. ZM: responsible for the assistance of animal experiments and methodology. DL: responsible for micro-CT and H&E staining. JS: funding acquisition, supervision, conceptualization, and formal analysis. TC: conceptualization, project administration, funding acquisition, methodology, and writing-review and editing. All authors contributed to the article and approved the submitted version.

## Conflict of Interest

The authors declare that the research was conducted in the absence of any commercial or financial relationships that could be construed as a potential conflict of interest.

## Supplementary Material

Supplementary Figure 1 Clinical pictures of A-PRF. **(A)** The appearance of A-PRF after eliminating the red blood cells. **(B)** A-PRF was compressed to a thin film by using a compression device. **(C)** A compression device of A-PRF.

Supplementary Figure 2 Histological and radiographic analysis of the whole sinus cavity at 1 and 4 weeks. **(A,B)** The tissue sections showed that the fracture of the basal bone corresponded to the perforated area of the SM (marked with black dotted line). **(C1–C2)** Micro-CT analysis of the maxillary sinus cavity at 4-week post-operation in CM and A-PRF groups.
