# Supplementary figures and images for: Histological and Histomorphometric Evaluation of Applying a Bioactive Advanced Platelet-Rich Fibrin to a Perforated Schneiderian Membrane in a Maxillary Sinus Elevation Model
Source: Front Bioeng Biotechnol. 2020 Nov 26;8:600032. doi: 10.3389/fbioe.2020.600032 (PMC7726256; doi:10.3389/fbioe.2020.600032)

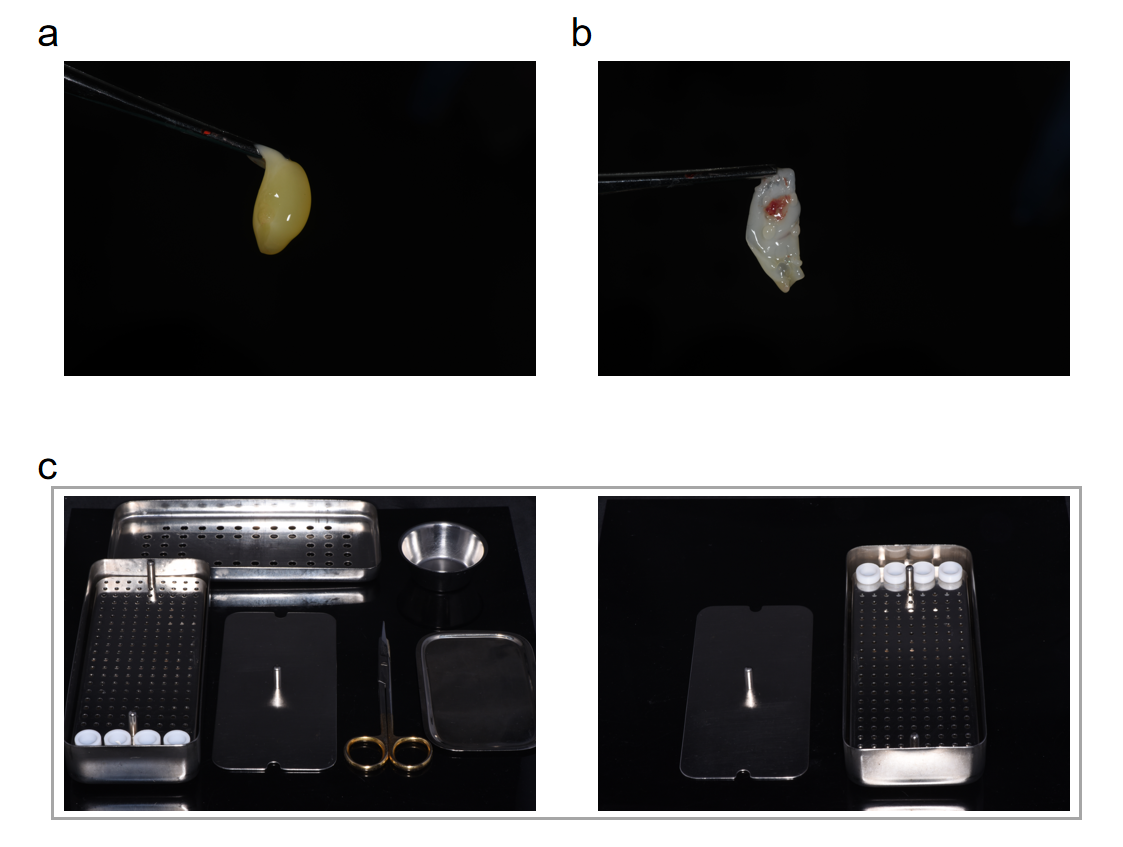

Supplement: Supplementary Figure 1 — Clinical pictures of A-PRF. (A) The appearance of A-PRF after eliminating the red blood cells. (B) A-PRF was compressed to a thin film by using a compression device. (C) A compression device of A-PRF. [file Image_1.TIF]

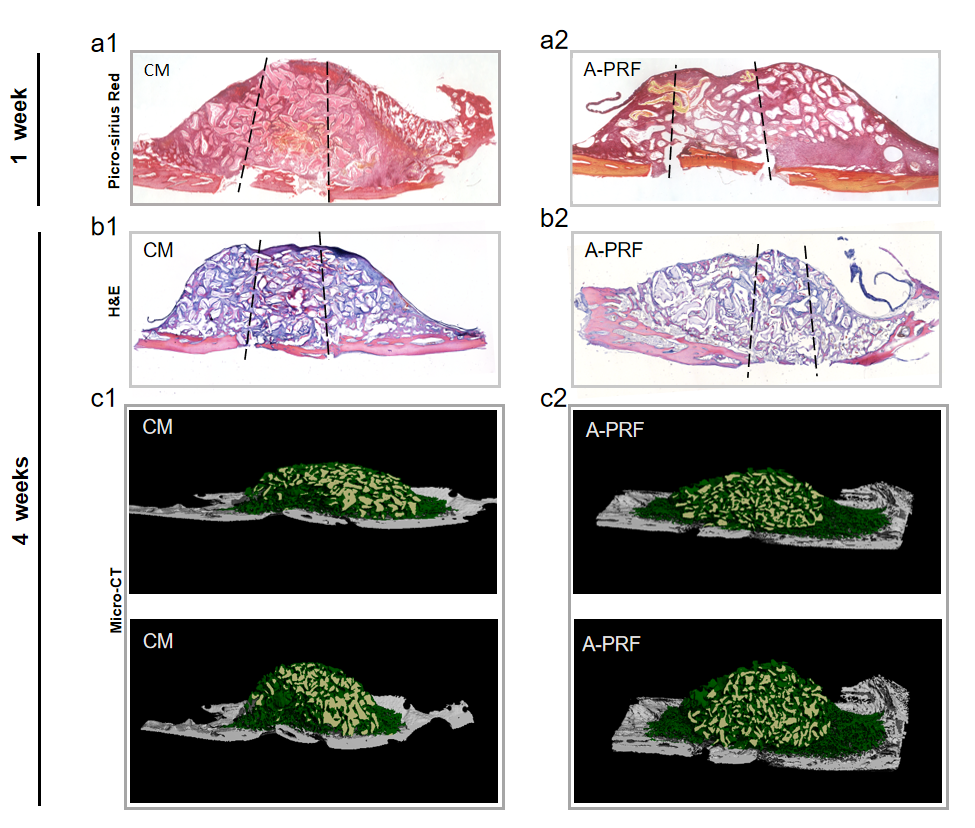

Supplement: Supplementary Figure 2 — Histological and radiographic analysis of the whole sinus cavity at 1 and 4 weeks. (A,B) The tissue sections showed that the fracture of the basal bone corresponded to the perforated area of the SM (marked with black dotted line). (C1–C2) Micro-CT analysis of the maxillary sinus cavity at 4-week post-operation in CM and A-PRF groups. [file Image_2.TIF]
